# Long-Term Outcome of Laparoscopic Sleeve Gastrectomy (LSG) on Weight Loss in Patients with Obesity: a 5-Year and 11-Year Follow-Up Study

**DOI:** 10.1007/s11695-023-06781-2

**Published:** 2023-08-22

**Authors:** Mohamed Abdul Moneim El Masry, Mostafa Abdel Megeed El Fiky

**Affiliations:** 1 Faculty of Medicine, Cairo University, PO Box 11562, Cairo, Egypt; 2 Military Production Specialized Medical Centre, 50 A Ismail Kamel St, Helwan, Cairo Egypt

**Keywords:** Obesity, Laparoscopic sleeve gastrectomy (LSG), Weight loss, Obesity-related complications resolution

## Abstract

**Background:**

Bariatric surgery is the most efficient treatment for patients with clinically severe obesity who have failed to obtain satisfactory weight loss through lifestyle modification and medical treatments. This study aimed to present our long-term laparoscopic sleeve gastrectomy (LSG) efficacy in terms of weight loss and obesity-related complications resolution.

**Patients and Methods:**

This is a retrospective study that was based on prospectively collected data from patients undergoing LSG by the same surgeon from July 2011 to the end of August 2022. The LSG-associated short-term (at 3 months, 6 months, and 1 year postoperatively) and long-term (at 5 years and 11 years postoperatively) weight loss, and the short-term (6 months postoperatively) and long-term (5 and 11 years postoperatively) rates of obesity-related complications were assessed.

**Results:**

This study included 892 patients who underwent LSG over 11 years. At the 1-year follow-up, data on 860 patients were available, while at the 5-year and 11-year follow-ups, data on 193 patients and 48 patients, respectively, were available. The mean EBWL% was 84.57 ± 18.41%, 64.22 ± 15.53%, and 66.01 ± 8.66% at the 1-year, 5-year, and 11-year follow-ups, respectively.

**Conclusion:**

This study adds new evidence concerning the short-term efficacy of LSG. The long-term assessment showed relatively sustainable weight loss and obesity-related complications resolution, with a regression of the short-term gains that was still far from the baseline burden.

**Graphical Abstract:**

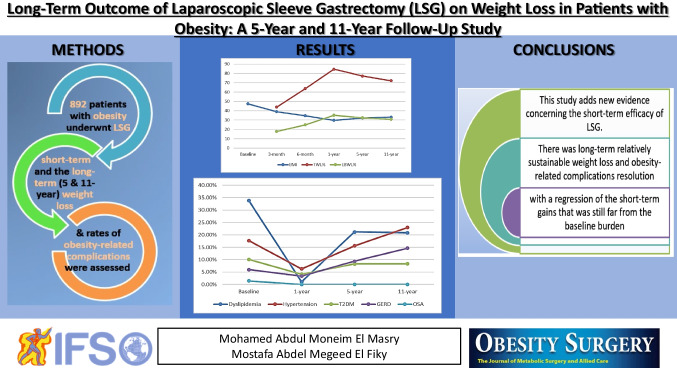

## Introduction

Obesity is a common disease that widely spreads as an epidemic all over the world. It is intimately related to several medical complications that, together, largely impact human health [1]. Bariatric surgery is the most efficient treatment for patients with clinically severe obesity who have failed to obtain satisfactory weight loss through lifestyle modification and medical treatments [2]. One of the current most commonly performed bariatric surgeries is laparoscopic sleeve gastrectomy (LSG). According to the 2018 IFSO survey, laparoscopic sleeve gastrectomy has been the most frequently performed bariatric procedure since 2014 [3], with a continually growing popularity that is attributed to its safety, efficacy, and technical simplicity[4].

In terms of the procedure’s efficacy, laparoscopic sleeve gastrectomy, like many other surgical solutions for severe obesity, has been demonstrated to be a successful treatment of obesity-associated metabolic disorders as well [2, 5]. Thus, it extends beyond being just a choice for weight loss to being a management procedure for several life-threatening conditions.

Indeed, the bariatric surgery efficacy could be only reliably identified at a long-term follow-up evaluation. In addition, the sustainability of obesity-related complications controls is yet to be confirmed in a long-term follow-up. To date, scarce evidence exists about long-term studies assessing the LSG outcome with more than 10 years of follow-up [6–11].

This study presents our long-term LSG efficacy in terms of weight loss and resolution of obesity-related complications.

## Patients and Methods

This is a retrospective study that was based on prospectively collected data from patients undergoing bariatric surgery. Consecutive patients planned for bariatric surgery at our institutions who underwent LSG by the same surgeon (Mohamed Abdul Moneim Amin El Masry) from July 2011 to the end of August 2022 were eligible for the study. The study was commenced after approval by the Research Ethics Committee and was conducted per the Declaration of Helsinki.

Patients with obesity were scheduled for bariatric surgery at the institutions of the study after a dedicated multidisciplinary assessment including complete history taking, clinical evaluation, and laboratory investigations. All patients underwent a preoperative assessment of gastroesophageal reflux disease (GERD) symptoms using a simple questionnaire published in 2011 [12] and an upper gastrointestinal (UGI) endoscopic assessment. Patients were candidates for the surgery if they fulfilled the criteria proposed by the field experts from international medical and surgical societies (International Federation for the Surgery of Obesity (IFSO), International Federation for the Surgery of Obesity—European Chapter (IFSO-EC), and European Association for the Study of Obesity (EASO)) [13–15].

The particular selection of patients for LSG rather than other bariatric techniques was based on the patient’s preference after a dedicated medical discussion with the surgeon, who clearly described each available choice’s benefits and potential shortcomings. Patients with evidence of severe GERD either established clinically (heartburn score ≥ 2, regurgitation score 3), or through a UGI endoscopic assessment (higher than grade B esophagitis, according to the Los Angeles (LA) classification [16], including erosive and Barrett’s esophagitis) and those with large hiatus hernia (> 4 cm as sized endoscopically by measuring the distance from the incisors to the top of the gastric folds and subtracting this figure from the corresponding measurement of the position of the crural pinch), were not candidates for LSG. Written informed consent was obtained from each patient before surgery. Patients with no available follow-up data on the hospital registry system were excluded.

The surgery was performed as previously described [17]. Briefly, after the standardized preoperative preparation, the surgery was performed under general anesthesia. Pneumoperitoneum was induced, and the sleeve was performed over a 36 Fr bougie with resection from the His angle to approximately 3–4 cm proximal to the pylorus. After surgery, routine postoperative care was provided. The patients were encouraged for early mobilization and received the postoperative diet and supplementation regimen and the follow-up visit schedules at 3, 6, and 12 months postoperatively, during which they were subjected to complete clinical assessments. They were informed to seek medical advice in case of any adverse event. The patients found having hiatus hernia had the hernia repaired through the mobilization of about 4 cm of the esophagus into the abdomen and approximating the crura posterior to the esophagus. Mesh was not needed since patients with large hiatus hernia were excluded.

Data concerning the patients’ demographics, anthropometric measurements, obesity-related complications, and surgery outcomes were recorded and analyzed.

Patients who had the operation performed 5 to 11 years ago were further contacted using the available telephone and address data on the hospital registry. Patients who could be reached were called for a follow-up visit in the outpatient clinic. Data from the responding patients was recorded and analyzed.

The percentages of the excess body weight loss (EBWL%) and the total weight loss (TWL%) were calculated as previously described [18]. The state of obesity-related complications was judged per the standardized outcome reporting published by the American Society for Metabolic and Bariatric Surgery [19]. GERD was considered based on the presence of typical symptoms and/or using proton pump inhibitor (PPI) therapy beyond the routinely prescribed postoperative use (for 3 months after surgery), according to the described guidelines [20].

### Study Outcomes

The study outcomes were the short-term (at 3 months, 6 months, and 1 year postoperatively), and the long-term (at 5 years and 11 years postoperatively) weight loss, and the short-term (6 months postoperatively) and the long-term (5 and 11 years postoperatively) rates of obesity-related complications.

### Statistical Analysis

Analysis of the patients’ data was performed using the SPSS statistical software (IBM Corp., Armonk, NY, USA), version 28. Categorical values were presented as frequencies and percentages, and the numerical data were presented as mean and standard deviation. A *p*-value of less than 0.05 was considered statistically significant.

## Results

**Fig. 1:**
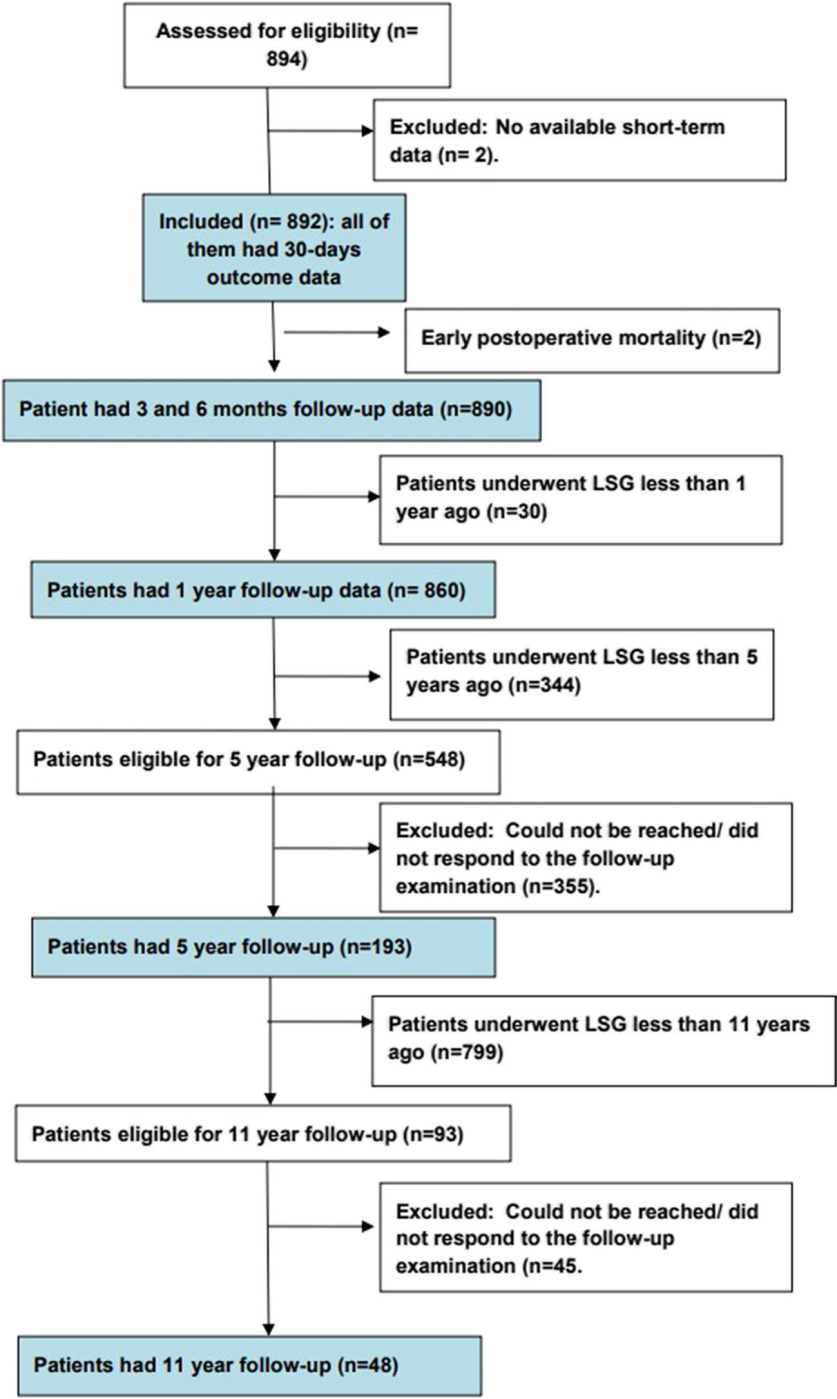
The patients’ flow chart

Initially, medical files of 894 patients could be retrieved for this retrospective study. Two patients were excluded due to unavailable short-term follow-up data, and then the study included 892 patients who underwent LSG over more than 11 years in multiple bariatric centers by the same surgeon. The patients’ flow chart is seen in Fig. 1. The patients’ ages ranged from 18 to 60 years, with a mean of 35.98 ± 10.25 years. There was a sex predilection toward females, who comprised 71.9% of the study patients. The baseline BMI ranged from 35.7 to 102 kg/m^2^, with a mean of 47.43 ± 7.57 kg/m^2^ (Table 1).

**Table 1:** Baseline demographic data of the study patients

		Study patients (*n* = 892)	
		Mean ± SD	Range
Age (year)		35.98 ± 10.25	18–60
Baseline weight (kg)		131.39 ± 25.26	80–270
Baseline BMI (kg/m^2^)		47.43 ± 7.57	35.7–102
EBW(kg)		71.1 ± 5.46	56.25–81
		Count	(%)
Sex	Male	251	28.1%
	Female	641	71.9%
Obesity-related complications			
Type 2 diabetes mellitus		90	10.1%
Hypertension		158	17.7%
Dyslipidemia		302	33.86%
Mild GERD		53	5.94%
Obstructive sleep apnea		13	1.45%
Smoking history			
Yes		74	8.3%
No		818	91.7%
UGI endoscopy			
Grade A esophagitis		31	3.48%
Grade B esophagitis		12	1.35%
Small hiatus hernia		6	0.67%

The most prevalent obesity-related complications were dyslipidemia (33.86%; *n* = 302), hypertension (17.7%; *n* = 158), and type 2 diabetes mellitus (10.1%; *n* = 90). Other complications were mild GERD (5.94%; *n* = 53) and obstructive sleep apnea (1.45%; *n* = 13) (Table 1).

### Short-Term Morbidity and Mortality

Early postoperative Clavien-Dindo grade III adverse events were encountered in 16 patients (1.79%). Six patients had intra-abdominal bleeding. Three of them were treated conservatively, and the other three patients indicated re-operation and hematoma drainage. One patient had an intra-abdominal leakage, which was managed by laparotomy with stent insertion. The patient, however, had acute sepsis, was admitted to the ICU, and died. Three patients had intraoperative bleeding and leakage. They were re-operated. One patient had an intra-abdominal leakage that was complicated by abscess formation and was subjected to laparoscopic exploration. One patient had wound hematoma and clinically suspected leakage. The patient was managed conservatively and received fresh frozen plasma and packed RBCs. There were another two cases of wound hematoma that were managed conservatively. One patient had wound bleeding, which was managed by percutaneous drain insertion. A massive pulmonary embolism occurred in one patient who was managed by anticoagulant and antithrombotic therapy and admitted to the ICU. The patient died in the ICU. Overall, two patients (0.22%) required ICU admission. The re-operation rate was 0.9% (*n* = 8), and the mortality rate was 0.22%.

#### Post-Surgery Follow-Up

The weight loss outcome was analyzed at the 3-month and 6-month follow-up visits for 890 patients since the 2 cases of early mortality were not included in the analysis.

The patients’ mean BMI was 39.05 ± 7.96 kg/m^2^ and 34.61 ± 6.96 kg/m2, the mean EBWL% was 43.95 ± 12.34% and 63.8 ± 15.55%, and the mean TWL% was 17.84 ± 4.31% and 24.91 ± 5.51% at the two time points, respectively (Fig. 1).

At the 1-year follow-up, data on 860 patients were available, while at the 5-year and 11-year follow-ups, data on 193/548 and 48/93 patients, respectively, were available.

**Fig. 2:**
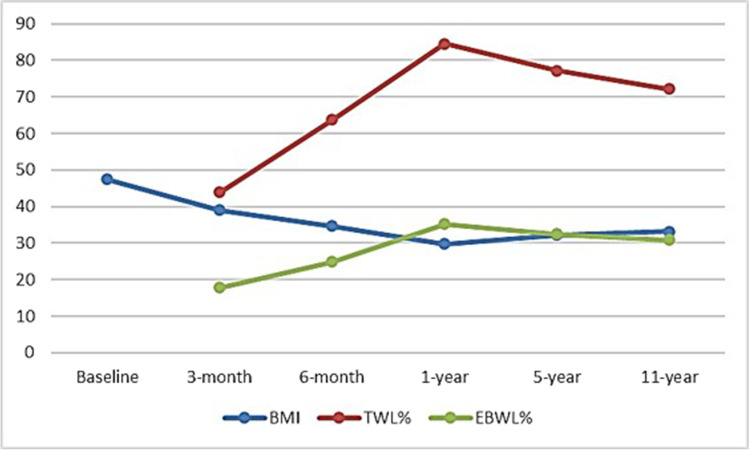
The patients’ weight loss outcome after surgery

The patients’ mean BMI at the 1-year follow-up was 29.76 ± 5.75 kg/m^2^, the mean EBWL% was 84.57 ± 18.41%, and the mean TWL% was 35.14 ± 6.51%. At the 5-year and 11-year follow-ups, the patients’ mean BMI was 32.24 ± 4.69 kg/m^2^ and 33.22 ± 5.28 kg/m^2^, the mean EBWL% values were 77.22 ± 15.3% and 72.15 ± 15.8%, and the mean TWL% values were 32.51 ± 4.17% and 30.91 ± 4.54%, respectively (Fig. 2). None of the patients exhibited insufficient weight loss at the 1-year follow-up. At the 5-year and 10-year follow ups, thirteen (6.74%) and seven (14.58%) patients were found to have recurrence in the weight gain, respectively. All patients with recurrence had sedentary lifestyle and the majority were sweat eaters (*n* = 17, 85%).

### Obesity-Related Complications Remission

**Fig. 3:**
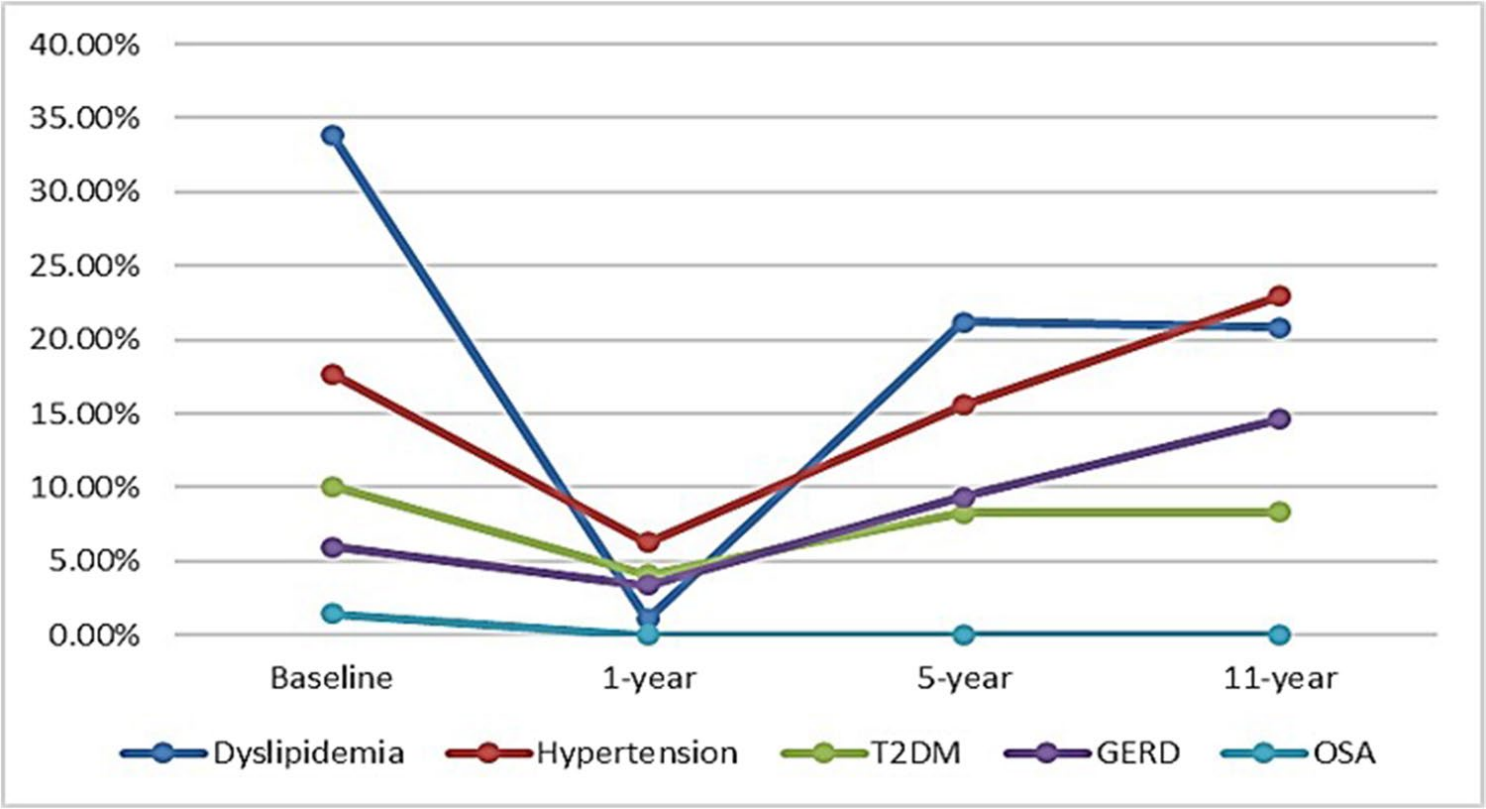
The patients’ obesity-related complications resolution outcome after surgery

At the 1 year postoperative follow-up, complete resolution occurred in 97.02% of patients with dyslipidemia (*n* = 293), 65.82% of patients with hypertension (*n* = 104), 61.11% of patients with diabetes mellitus (*n* = 55), and 100% of patients with obstructive sleep apnea (*n* = 13). There was an improvement in 27 patients with hypertension (17.09%) and in 11 patients with diabetes mellitus (12.22%). Concerning GERD, symptomatic relief occurred in 37 patients (69.81%) who described only sporadic use of PPIs (2–3 times per month), with their UGI endoscopy revealing normal findings in 30 patients and grade A esophagitis in 7 patients. On the other hand, worsening in GERD occurred in 3 patients (5.66%), who were confirmed by endoscopy to have grade C esophagitis, of whom two patients were found having erosive esophagitis resistant to conservative management and underwent conversion to RYGB (0.45%). De novo mild GERD symptoms were experienced by 13 patients. Thus, overall, the postoperative GERD rate was 3.25% (29 patients) (Fig. 3).

At the 5-year postoperative follow-up, the rates of dyslipidemia, hypertension, T2DM, and GERD were 21.2% (41/193), 15.6% (30/193), 8.29% (16/193), and 9.32% (18/193), respectively. At the 11-year postoperative follow-up, the rates were 20.83% (10/48), 22.92% (11/48), 8.33% (4/48), and 14.58% (7/48), respectively (Fig. 3). The long-term changes in obesity-related complications, as compared to the achieved improvement at the 1-year follow-up, are shown in Table 2.

**Table 2:** Changes in obesity-related complication at the long-term follow-up

		Resolution	Improvement	Unchanged	Worsening	Denovo cases	Total cases with disease
Dyslipidemia	5-year follow-up	0	0	3	0	38	41
	11-year follow up	0	1	4	1	4	10
Hypertension	5-year follow-up	2	1	22	0	7	30
	11-year follow up	0	1	6	0	4	11
T2DM	5-year follow-up	0	0	11	0	5	16
	11-year follow up	0	0	9	0	2	11
GERD	5-year follow-up	0	0	7	0	4	11
	11-year follow up	0	0	2	1	1	4

Concerning the six patients with preoperative, at the 1-year follow-up, four of them had relieved GERD symptoms, one patient had persistent mild GERD with grade A esophagitis, and one showed hernia recurrence and aggravation of GERD symptoms. However, the patient continued on the conservative treatment. Only one patient could be reached at the long-term follow up (at 5-year), with no GERD symptoms or hernia recurrence.

The mean HbA1c (%) levels for the patients with preoperative diabetes significantly dropped from 8.65 ± 0.67 to 6.48 ± 1.6 at the 1-year follow-up, 6.83 ± 1.91 at the 5-year follow-up, and 6.77 ± 1.72 at the 11 year follow-up (*p* < 0.001).

## Discussion

This study presents a single-surgeon experience over a period of more than 11 years, including 892 patients after LSG. Since most studies report the outcome of LSG at 1, 2, or 3 years of follow-up as a maximum, it is believed that the long-term outcome of LSG, namely the durability of the weight loss and remission of obesity-related complications, still needs to be supported. The fast evolution of LSG with its promising short-term outcomes has likely impacted the full assessment of long-term efficacy. To the best of our knowledge, this is one of a few studies in the literature with more than 10 years of follow-up after LSG [6–11].

In the current work, the peak of weight loss was shown at the end of the first year postoperatively (84.57 ± 18.41%). The patients who completed the 5-year follow-u and those who completed the 10-year follow-up showed obviously lower EBWL% at 77.22 ± 15.3% and 72.15 ± 15.8%, denoting regression in the weight loss in the 5- and 10-year follow-up.

Comparable to our results, Arman et al. reported a mean short-term EBWL% of 82.4% that was reduced at the long-term follow-up (at 11 years) [7]. Almost all studies that assessed the long-term weight loss after LSG reported a decline in EBWL% after a short-term peak [6, 8–11, 21, 22]. However, like this study, the recurrence of weight gain was mild in most cases according to the threshold reported by Sakran et al. (< 15–30% of the EBW) [9]. The long-term recurrence of weight gain that occurs after LSG is multifactorial, with several potentially influencing factors such as genetic basis, culture, lifestyle, dietary patterns, and an expanded sleeve over time [23, 24].

Despite the initial thought that LSG is merely a restrictive procedure, evidence concerning its metabolic effect currently exists. This was supported in the present study by the postoperative remarkable amelioration of the associated medical complications. Likewise, Kraljević et al. and Sakran et al. reported that LSG provided a meaningful resolution of obesity-related complications [8, 9].

The partial regression of the initial achievements that followed LSG was also shown in the context of obesity-related complications remission. Assessment of the counterpart rates in patients who had the surgery performed 5 and 11 years before showed higher rates of the described complications, apart from obstructive sleep apnea which was absent in the assessed patients. Despite higher levels of dyslipidemia, hypertension, and T2DM at the long-term evaluation, these complication rates did not reach the baseline preoperative levels. These data denote that the maximum improvement occurs at the 1-year follow-up, after which some decline occurs. As for dyslipidemia, this high number of de novo cases is plausibly explained by the recurrence of weight gain, older age, and maintaining a poor lifestyle (including sedentary life and unhealthy food), which was the cause of obesity in these patients.

A strong association between GERD and obesity exists. GERD is the major factor contributing to the impairment of the quality of life, with possible effects on the patient’s physical and mental functioning [25, 26].

GERD assessment in the present work revealed resolution, worsening, persistence, and de novo occurrence, with overall fewer GERD cases in the short-term compared to the baseline, however, with two cases of conversion to RYGB due to refractory GERD.

The GERD state after LSG remains a matter of controversy. Many researchers presumed that LSG precipitates GERD symptoms [22, 27–30]. De novo LSG-related occurrence or the worsening of GERD could be explained, as previously stated, by the surgery-related disruption of the His angle, partial division of the lower esophageal sphincter sling fibers, restricted stomach cavity with reduced compliance, or antral dysfunction [31]. Factors contributing to the resolution of GERD in this study are likely attributed to the postoperative decrease in abdominal fat with a subsequent reduction of the intra-abdominal pressure and a decrease in the production of gastric acid due to resection of the fundus, and acceleration of gastric emptying [32].

The long-term assessment in this study demonstrated an increase in the rate of GERD, which is consistent with the reported increase in patients suffering from GERD with a longer follow-up period [10, 11, 33].

Based on this study’s findings, the LSG’s efficacy, namely the weight loss outcome and obesity-related complications resolution, was evident in the short-term, with mild regression in the long run.

The study has a number of limitations. First is the retrospective design with high rates of patient loss at the long-term follow-up. Second, the postoperative GERD cases were mainly assessed clinically based on symptoms and PPI intake. The study, however, is one of a few assessing LSG patients after more than 10 years.

## Conclusion

This study adds new evidence concerning the short-term efficacy of LSG. The long-term assessment showed relatively sustainable weight loss and obesity-related complications resolution, with a regression of the short-term gains that was still far from the baseline burden.

## Data Availability

The datasets analyzed during the current study are available upon an editorial request.
